# Effects of isometric handgrip training on blood pressure among hypertensive patients seen within public primary healthcare: a randomized controlled trial

**DOI:** 10.1590/1516-3180.2020.0796.R1.22042021

**Published:** 2021-11-15

**Authors:** Aline Cabral Palmeira, Breno Quintella Farah, Gustavo Oliveira da Silva, Sérgio Rodrigues Moreira, Mauro Virgílio Gomes de Barros, Marilia de Almeida Correia, Gabriel Grizzo Cucato, Raphael Mendes Ritti-Dias

**Affiliations:** I MSc. Professor, Physiotherapy and Nursing Departments, Faculdade São Francisco de Juazeiro (FASJ), Juazeiro (BA), Brazil.; II PhD. Professor, Physical Education Department, Universidade Federal Rural de Pernambuco, Recife (PE), Brazil; Associate Researcher, Postgraduate Program on Physical Education, Universidade Federal do Pernambuco (UFPE), Recife (PE), Brazil.; III MSc. Doctoral Student, Postgraduate Program on Rehabilitation Sciences, Universidade Nove de Julho (UNINOVE), São Paulo (SP), Brazil.; IV PhD. Professor, Postgraduate Program on Physical Education, Universidade Federal do Vale do São Francisco (UNIVASF), Petrolina (PE), Brazil.; V PhD. Professor, Postgraduate Program on Physical Education, Universidade de Pernambuco (UPE), Recife (PE), Brazil.; VI PhD. Professor, Postgraduate Program on Medicine, Universidade Nove de Julho (UNINOVE), São Paulo (SP), Brazil.; VII PhD. Professor, Department of Sport, Exercise and Rehabilitation, Northumbria University, Newcastle upon Tyne, United Kingdom.; VIII PhD. Professor, Postgraduate Program on Rehabilitation Sciences, Universidade Nove de Julho (UNINOVE), São Paulo (SP), Brazil.

**Keywords:** Hypertension, Resistance training, Primary health care, Blood pressure, Cardiac autonomic modulation, Strength training, Primary care, Isometric

## Abstract

**BACKGROUND:**

Meta-analyses have demonstrated that isometric handgrip training (IHT) decreases blood pressure in hypertensive individuals. Nonetheless, most studies were conducted in laboratory settings and its effects in real-world settings remain unclear.

**OBJECTIVE:**

To analyze the eﬀects of IHT on office and ambulatory blood pressure in hypertensive patients attended within primary healthcare.

**DESIGN AND SETTING:**

Randomized controlled trial conducted in primary healthcare units within the Family Health Program, Petrolina, Pernambuco, Brazil.

**METHODS:**

63 hypertensive patients (30-79 years old; 70% female) were randomly allocated into IHT or control groups. IHT was performed three times per week (4 x 2 minutes at 30% of maximal voluntary contraction, one-minute rest between bouts, alternating the hands). Before and after the 12-week training period, office and ambulatory blood pressure and heart rate variability were obtained. The signiﬁcance level was set at P < 0.05 (two-tailed testing) for all analyses.

**RESULTS:**

IHT signiﬁcantly decreased office systolic blood pressure (IHT: 129 ± 4 versus 121 ± 3 mmHg, P < 0.05; control: 126 ± 4 versus 126 ± 3 mmHg, P > 0.05), whereas there was no effect on diastolic blood pressure (IHT: 83 ± 3 versus 79 ± 2 mmHg, P > 0.05; control: 81 ± 3 versus 77 ± 3 mmHg, P > 0.05). Heart rate variability and ambulatory blood pressure were not altered by the interventions (P > 0.05 for all).

**CONCLUSION:**

IHT reduced office systolic blood pressure in hypertensive patients attended within primary care. However, there were effects regarding diastolic blood pressure, ambulatory blood pressure or heart rate variability.

**CLINICALTRIALS.GOV IDENTIFIER:**

NCT03216317.

## INTRODUCTION

Hypertension impacts over one billion people worldwide and is the main risk factor for heart and cerebrovascular diseases, accounting for 13% of global deaths.^1–3^ The therapeutic approach for hypertensive patients includes drug therapy and lifestyle changes in association with drug therapy, with the aim of reducing blood pressure (BP) to the target normal range (< 130/80 mmHg).^2^

Previous meta-analyses have shown that isometric handgrip training (IHT) decreases office BP in hypertensive patients by more than 5 mmHg after a few weeks.^4–9^ The American College of Cardiology and the American Heart Association have recently recommended IHT as a potential alternative strategy for lowering BP, but with a low level of evidence.^10^

From a clinical point of view, reductions in BP are relevant when this impacts on BP levels during a major part of the time. Interestingly, the effects of IHT on ambulatory BP, which is more related to cardiovascular events than office BP, have not been demonstrated. In fact, in three previous studies, despite significant reductions in office BP, no effects on ambulatory BP were shown among hypertensive individuals after IHT, thus suggesting that there was a need for further studies.^11–13^

The benefits of IHT comprise its ease of application and the short time needed for doing the exercise. Therefore, it is ideal for application within primary care, in non-laboratory settings. However, all clinical trials studies analyzing the effects of IHT on BP were conducted either in laboratory^14^ or in home settings.^11,15^ The potential effectiveness of this type of training at primary healthcare units is therefore unknown. Primary healthcare is the first point of contact that people have with the healthcare system when they have a health problem. The healthcare services provided within primary care include treatment of health conditions and support for managing long-term healthcare, including chronic conditions such as hypertension, at lower cost than in hospital settings.

## OBJECTIVE

In this study, we analyzed the effects of IHT on office and ambulatory BP in hypertensive patients attended at a primary healthcare unit. Our hypothesis was that IHT would reduce BP similarly in non-laboratory settings.

## METHODS

### Experimental approach to the problem

A randomized controlled trial was used to investigate the effects of IHT on office and ambulatory BP among hypertensive patients attended at a primary healthcare unit. Medicated hypertensive patients were randomly assigned to either the IHT group or the control group. Ambulatory BP, office BP and heart rate variability parameters were measured before and after the 12-week intervention period by researchers blinded to the group allocations.

### Trial design

This randomized controlled trial followed the Consolidated Standards of Reporting Trials (CONSORT) and was registered in the www.clinicaltrials.gov database under the registration number NCT03216317 and formed part of the ISOPRESS network.^16,17^ The study methods were approved by the Institutional Review Board of Universidade Federal do Vale do São Francisco (protocol number: 61442216.5.0000.5196; approval date: May 16, 2017) in conformity with the national research ethics system guidelines and with the Helsinki Declaration of 1975 (revised in 1983). Before participation, subjects provided written informed consent.^18^

### Subjects

We invited medicated hypertensive patients at primary care units within the Family Health Program in the city of Petrolina, state of Pernambuco, northeastern Brazil, to participate in this study. These primary care units form part of the Brazilian public healthcare system, which serves the population in places near patients’ homes. The eligibility criteria for the study were that the subjects needed to: i) be using anti-hypertensive medications; ii) be over the age of 18 years old; iii) have no presence of diabetes or cardiovascular disease (other than hypertension); iv) have no limitations on undergoing isometric handgrip training; and v) not be engaged in any systematic exercise programs assessed through the International Physical Activity Questionnaire. The exclusion criteria were any of the following situations: (a) changes to the type or dose of blood pressure control medicine; (b) engaging in another exercise program; or (c) taking part in less than 80% of the isometric handgrip training sessions.

### Randomization and allocation

The participants were block-randomized using a random number table (using the website https://www.randomizer.org), with stratiﬁcation according to sex and baseline office systolic BP (done by a researcher who did not participate in the subject recruitment or data collection), into two groups: IHT group and control group. The allocation information was concealed from the researchers performing the measurements.

### Interventions

The patients allocated to the IHT group trained three times per week, for a total of 12 weeks, in healthcare units that form part of the Family Health Program. Each session was composed of four sets of two-minute isometric contractions (alternating the hands), done through a handgrip dynamometer (Zona Health, Boise, Idaho, United States) at 30% of each patient's maximal voluntary contraction, which was established at the start of each session via the handgrip dynamometer. The Zona Plus dynamometer was developed specifically for isometric handgrip training. The screen in the device provides instantaneous feedback of the amount of force and indicates whether the amount of force applied is sufficient for the intensity selected. In addition, the device has a timer that provides information regarding the duration of the exercise and the rest intervals. Patients allocated to the control group were encouraged to increase their level of physical activity, but with no particular guidance on physical activity.

### Measurements

Cardiovascular variables were measured at the baseline and at a follow-up (12 weeks later). The participants received the following instructions for what they should do before the cardiovascular evaluations: (a) have a light meal prior to arrival at the laboratory; (b) refrain from moderate-to-vigorous physical activity for at least 24 h before to the visit; and (c) refrain from smoking or alcohol or caﬀeine consumption for at least 12 h. Researchers who were blinded to the group allocations collected the data. The post-intervention evaluation was performed at least 72 hours after the last exercise session.

#### Office BP:

The office BP was measured through the Omron HEM 742 device (Omron Healthcare, Kyoto, Japan). After 10 minutes of supine rest, at least three consecutive measurements with one-minute intervals between them were assessed. The measurements were made on the right arm, with an appropriate cuﬀ size for the arm circumference.^19^ The intraclass correlation coeﬃcient for systolic BP was 0.85, and for diastolic BP it was 0.92.^20^

#### Ambulatory BP:

The ambulatory BP was obtained through an oscillometric device (Dyna-MAPA, Cardios, Brazil) that had previously been set up for performing BP assessments every 15 minutes during the daytime period and every 30 minutes during the nighttime, based on previously reported procedures.^21^ Also, patients were counseled to report crucial everyday activities, such as meals, movement from one place to another and medications.

#### Heart rate variability:

The heart rate variability was evaluated from the RR intervals, measured through a heart rate monitor (Polar V800, Polar Electro, Kempele, Finland) in the supine position for 10 minutes. At least five minutes of stationary R-R interval data were analyzed. All analyses were carried out by a single experienced evaluator who was blind to the group allocations. The intraclass correlation coefficient for this evaluator spanned from 0.990 to 0.993.^22^ All heart rate variability analysis procedures followed previously described guidelines.^23^ The Kubios HRV software (Biosignal Analysis and Medical Imaging Group, Joensuu, Finland) was used for the analysis. The time (standard deviation of all RR intervals [SDNN], root mean square of the squared differences between adjacent normal RR intervals [RMSSD] and percentage of adjacent intervals over 50 ms [PNN50]) and frequency (low frequency component, high frequency component and sympathovagal balance) domain variables were obtained.

### Statistical analyses

To determine the sample size, we used previously demonstrated data on the mean reduction and standard deviation (SD) of office systolic BP following IHT.^24^ Given an expected reduction of 6.0 ± 4.6 mmHg and α of 0.05 and β of 0.20, an estimated sample size of 28 participants (14 per group) was deemed sufficient.

Normality and homogeneity of variances were verified by means of the Shapiro-Wilk test and the Levene test, respectively. Clinical characteristics were compared between the groups using the t test, chi-square test and Fisher test. To analyze the effects of isometric handgrip training on BP, generalized estimating equations were used, along with post-hoc pairwise comparison using the Bonferroni correction for multiple comparisons. Effect size (ES) was used to stipulate the magnitude of differences in the same group. Intention-to-treat analysis was used to estimate overall effects, among all the randomized patients while ignoring noncompliance and dropouts, and the data were imputed with linear regression weighted according to group. The signiﬁcance level was set at P < 0.05 (two-tailed testing) for all analyses. The data were presented as means and standard errors or as 95% confidence intervals. Categorical variables were summarized as relative frequencies.

## RESULTS

**Figure 1:**
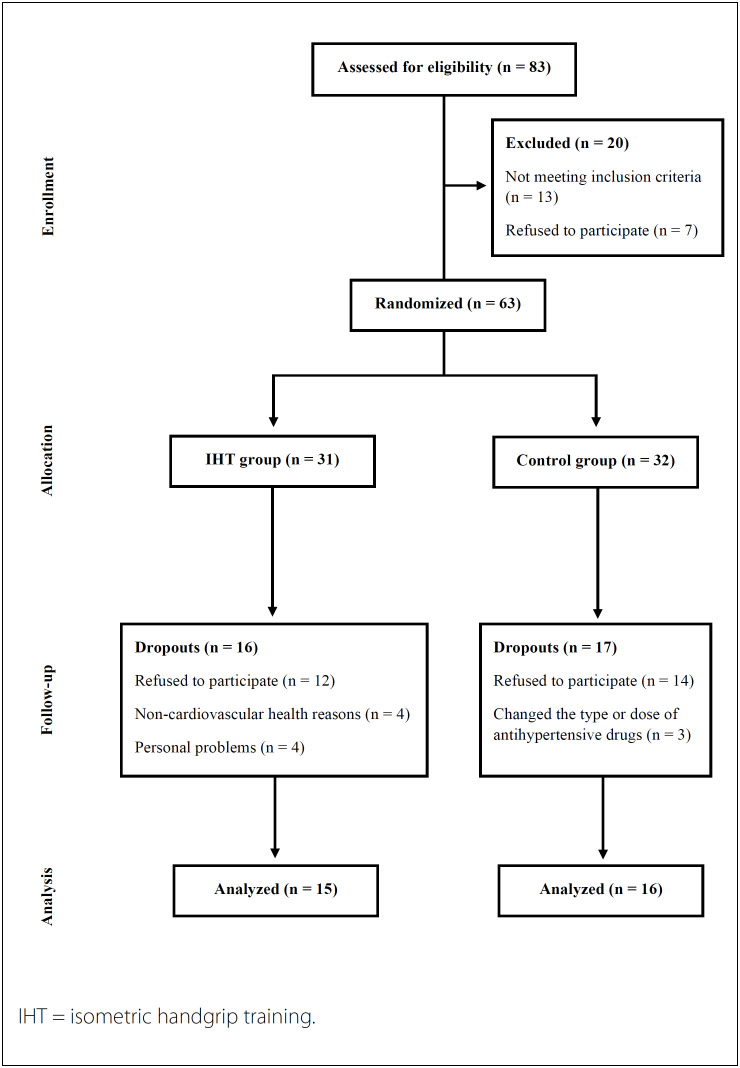
Flowchart of study.

The recruitment and intervention periods encompassed July 2017 July to July 2018. The study ﬂowchart is shown in Figure 1. The groups were similar at the baseline (Table 1).

**Table 1:** General characteristics of experimental groups at baseline

Variables	IHT	Control group	P
Age (years)	54.3 ± 3.7	52.7 ± 2.6	0.743
Weight (kg)	74.1 ± 3.2	80.1 ± 4.9	0.319
Body mass index (kg/m²)	29.4 ± 1.1	31.6 ± 1.5	0.247
Office systolic BP (mmHg)	129 ± 4	126 ± 4	0.241
Office diastolic BP (mmHg)	83 ± 3	82 ± 3	0.632
Walking (minutes/week)	122 ± 25	73 ± 17	0.123
Moderate physical activity (minutes/week)	135 ± 35	64 ± 26	0.116
Sex (% men)	27	31	0.909
Current smoker (%)	18.8	0	0.103
Calcium channel blocker (%)	7	6	0.898
Diuretic (%)	73	56	0.290
ß-blocker (%)	20	11	0.478
ACE inhibitor (%)	20	17	0.805
Angiotensin receptor blockers (%)	67	78	0.475

Values that are not percentages are presented as mean ± standard error. IHT = isometric handgrip training; BP = blood pressure.

The dropout rates were 51.6% in the isometric handgrip training group and 53.1% in the control group. Through comparing the characteristics of the patients who were included and the dropouts in the isometric handgrip training group (Table 2), only a difference in calcium channel blocker use could be seen (P < 0.05). One 61-year-old woman in the isometric handgrip training dropped out due to joint pain. Adherence in the IHT group was 84.6% (95% confidence interval, CI: 82.2% to 87.1%).

**Table 2:** Comparison of the characteristics of the patients who were included and who were dropouts in this study

Variables	Included n = 31	Dropout n = 33	P
Intervention group (%)	45.7	53.3	0.540
Age (years)	53.6 ± 2.2	55.6 ± 1.8	0.417
Weight (kg)	75.6 ± 2.6	75.1 ± 2.8	0.890
Body mass index (kg/m²)	30.1 ± 0.9	29.1 ± 0.9	0.431
Office systolic BP (mmHg)	126 ± 3	128 ± 2	0.518
Office diastolic BP (mmHg)	81 ± 2	79 ± 2	0.401
Walking (minutes/week)	97 ± 15	157 ± 66	0.366
Moderate physical activity (minutes/week)	99 ± 22	99 ± 22	0.165
Sex (% men)	27	50	0.183
Calcium channel blocker (%)	3	33	0.002
Diuretic (%)	64	40	0.079
ß-blocker (%)	15	27	0.259
ACE inhibitor (%)	18	20	0.854
Angiotensin receptor blockers (%)	73	60	0.285

Values that are not percentages are presented as mean ± standard error. IHT = isometric handgrip training; BP = blood pressure.

**Figure 2:**
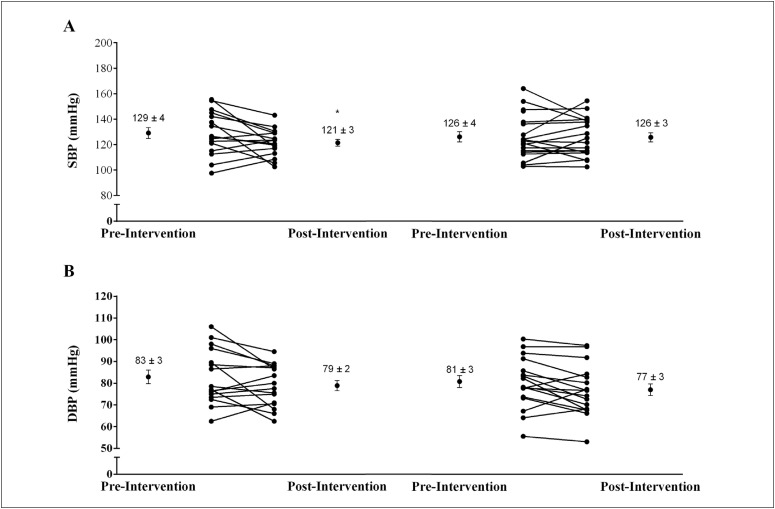
Effects of isometric handgrip training on office blood pressure. A – Systolic blood pressure (SBP) (P-values: group = 0.900; time = 0.088; GxT = 0.049); B – Diastolic blood pressure (DBP) (P-values: group = 0.531; time = 0.003; GxT = 0.933). *Signiﬁcant difference from Pre (P < 0.05). IHT, isometric handgrip training; CG, control group; GxT, group-time interaction.

Figure 2 and Table 3 present the effects of IHT on office BP and heart rate variability parameters, respectively. A group-time (GxT) interaction was observed for office systolic BP (power = 0.83), which indicated that only the group that performed isometric handgrip training presented reductions in office systolic BP (IHT: 129 ± 4 versus 121 ± 3 mmHg; and control: 126 ± 4 versus 126 ± 3 mmHg; P < 0.05). No GxT interaction was observed in relation to office diastolic BP (IHT: 83 ± 3 versus 79 ± 2 mmHg; and control: 81 ± 3 versus 77 ± 3 mmHg; P > 0.05) (power = 0.52) and heart rate variability parameters (P > 0.05 for all).

**Table 3:** Effects of isometric handgrip training on heart rate variability parameters in hypertensive individuals

Variables	IHT		Control		P
	Pre-Intervention	Post-Intervention	Pre-Intervention	Post-Intervention	
RR interval (ms)	819 ± 32	854 ± 23	831 ± 22	829 ± 25	0.166
SDNN (ms)	31.6 ± 4.5	32.4 ± 5.6	34.3 ± 2.9	34.7 ± 3.3	0.928
RMSSD (ms)	29.1 ± 7.5	32.3 ± 8.8	25.1 ± 2.9	27.0 ± 3.4	0.923
PNN50 (%)	9.2 ± 4.6	11.8 ± 4.7	7.4 ± 2.3	8.5 ± 2.7	0.837
LF (nu)	47.9 ± 4.7	50.0 ± 5.6	57.3 ± 5.1	56.5 ± 4.9	0.595
HF (nu)	51.5 ± 4.7	49.8 ± 5.6	42.5 ± 5.1	43.3 ± 4.9	0.664
LF/HF	1.17 ± 0.18	1.44 ± 0.311	2.07 ± 0.39	2.32 ± 0.76	0.785

Values are presented as mean ± standard error. IHT = isometric handgrip training; HF = High frequency; LF = Low frequency; SDNN = standard deviation of all RR intervals; RMSSD = root mean square of the squared differences between adjacent normal RR intervals; PNN50 = percentage of adjacent intervals over 50 ms; LF/HF = sympathovagal balance; nu = normalized units.

**Figure 3:**
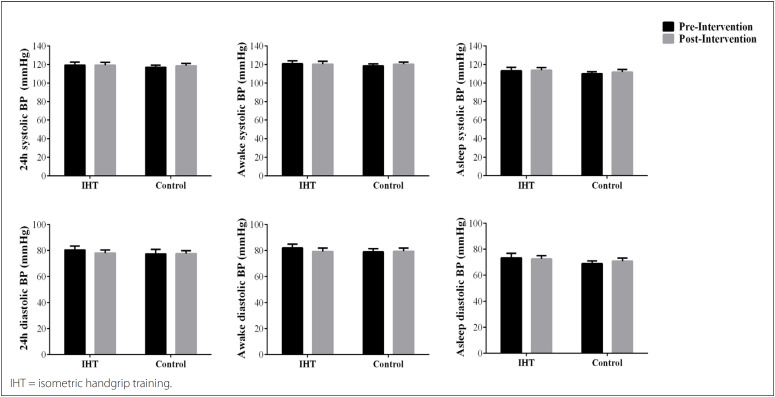
Effects of isometric handgrip training on ambulatory blood pressure.

Figure 3 presents the effects of IHT on ambulatory BP. No group-time interaction (P > 0.05 for all) was observed for BP, overall over a 24-hour period (systolic BP: IHT 119.2 ± 3.3 versus 119.2 ± 3.0 mmHg, ES = 0.01; control 116.9 ± 2.2 versus 118.6 ± 2.4 mmHg, ES = 0.18, power = 0.54; diastolic BP: IHT 80.5 ± 3.0 versus 78.1 ± 2.4 mmHg, ES = 0.27; control 77.5 ± 2.3 versus 77.7 ± 2.2 mmHg, ES = 0.02, power = 0.69); or while the subjects were awake (systolic BP: IHT 120.7 ± 3.3 versus 120.3 ± 2.9 mmHg, ES = 0.03; control 118.4 ± 2.2 versus. 120.1 ± 2.3 mmHg, ES = 0.18; diastolic BP: IHT 82.0 ± 2.9 versus 79.3 ± 2.5 mmHg, ES = 0.27; control 79.0 ± 2.4 versus 79.5 ± 2.3 mmHg, ES = 0.05) or asleep (systolic BP: IHT 113.1 ± 3.7 versus 113.8 ± 2.8 mmHg, ES = 0.06; control 110.0 ± 2.1 versus 111.7 ± 2.7 mmHg, ES=0.17; diastolic BP: IHT 73.3 ± 3.6 versus 72.5 ± 2.4 mmHg, ES = 0.07; control 69.0 ± 2.1 versus 70.8 ± 2.3 mmHg, ES = 0.20) (Figure 3).

The intent-to-treat analysis did not reveal any significant effect from the IHT program on any of the outcome variables measured (data not shown).

## DISCUSSION

The main results of this study in a primary healthcare unit were the following: (i) IHT reduced office systolic BP among medicated hypertensive individuals; (ii) no effects were observed in relation to office diastolic BP, heart rate variability or ambulatory BP in medicated hypertensive patients.

The main novelty of this study was that the IHT program was conducted in a primary healthcare unit, which is a real-world setting for supervised training. We demonstrated that there was a reduction in office systolic BP in medicated hypertensive individuals, which corroborates previous studies conducted in laboratory or home settings.^11,14,15^ The magnitude of the reduction in office systolic BP was approximately 8 mmHg, which was similar to findings from previous clinical trial studies conducted in laboratory or home settings.^8,25^ Moreover, the analysis on individual responses indicated that 63% of the patients showed reductions in systolic BP of more than 5 mmHg, which may represent a reduction of at least 7% in the risks of stroke, coronary disease and death.^26^ Thus, IHT may be incorporated as alternative strategy for controlling office systolic BP in medicated hypertensive individuals who are treated in a primary care unit.

On the other hand, 12 weeks of IHT performed in a primary healthcare unit did not change office diastolic BP in these medicated hypertensive individuals. Although this result contrasts with the findings from some studies, there are other studies that also reported that there was no reduction in diastolic BP after IHT, among hypertensive patients.^4–7^ After six weeks of IHT in a laboratory setting, Peters et al. did not observe any reduction in office diastolic BP, although they observed a reduction in office systolic BP.^27^ Similarly, Taylor et al. demonstrated that there was a decrease in office systolic BP, but not in diastolic BP, after 10 weeks in a laboratory setting.^28^ Lastly, after 12 weeks of IHT in a laboratory or home setting, Gordon et al. did not find any reduction in diastolic BP.^15^

It is not clear why office systolic BP, but not diastolic BP, was found to have decreased after the 12-week training period. One possible explanation is that the patients included in the present study presented well-controlled values for diastolic BP in the pre-intervention period (all < 90 mmHg) and, as such, may have had a lower capacity for BP reduction through IHT. In fact, a previous study reported that individuals with higher pre-training resting BP had a greater post-training hypotensive response.^29^

In the present study, 12 weeks of IHT in hypertensive individuals did not have the capacity to promote improvement in cardiac autonomic modulation to the heart. Farah et al. did not find any improvements after 12 weeks of supervised or home-based isometric handgrip training in hypertensive individuals who were using medications. Similarly, Stiller-Moldovan et al. did not find any changes in cardiac autonomic modulation after 8 weeks of isometric handgrip training in hypertensive patients.^11,13^ In contrast, Taylor et al. found improvements in high-frequency spectral power among uncontrolled hypertensive individuals after supervised isometric handgrip training. Interestingly, in Taylor's study, the baseline BP values were higher than those of the present study (156 versus 129 mmHg). This indicates that isometric handgrip training might lead to improvements in autonomic cardiac modulation in individuals with uncontrolled BP.^28^ Therefore, it is possible that other mechanisms are involved in the reduction of BP after IHT.^30^

Recently, our group demonstrated^31^ that a reduction in arterial stiffness occurs in hypertensive individuals who are responsive to isometric handgrip training. In addition, Peters et al. showed that there was enhancement of oxidative stress after six weeks of training and McGowan et al. observed improvement in endothelial function after eight weeks of IHT.^27,31,32^

Ambulatory BP has been considered more important than office BP, in terms of clinical perspective, since it presents better prediction of target organ damage and cardiovascular mortality.^33^ Our results indicated that there was no reduction in any of the ambulatory BP measurements after 12 weeks of IHT performed in a primary healthcare unit and are in agreement with previous studies.^11–13^ A study conducted by Stiller-Moldovan et al. did not observe any reduction in ambulatory BP after eight weeks of isometric exercise training performed at 30% of maximal voluntary contraction.^13^ Moreover, Pagonas et al. also showed that there was no reduction in ambulatory BP after 12 weeks of handgrip exercise training performed five times per week at 30% of maximal voluntary contraction, in a hypertensive population.^12^ Therefore, these results indicate that isometric handgrip training presents only a transient effect on BP that is only observed in office BP and is not prolonged during ambulatory activities.

The American Heart Association and American College of Cardiology have recommended isometric handgrip training as a potential alternative strategy for lowering BP in the hypertensive population.^10^ The benefits of this type of training comprise its ease of application and the short time that needs to be dedicated to implementing the exercise, such that it is ideal for application in primary care and in non-laboratory settings. In fact, three sessions per week and 12 minutes per session (i.e. 36 minutes per week) is less time than the current recommendations^34,35^ for physical exercise (150 minutes per week), which therefore enables avoidance of important barriers to physical activity practice among patients with cardiovascular diseases.^36,37^

In the present study, we demonstrated that isometric handgrip training reduces office systolic BP in medicated hypertensive patients who were attended in primary healthcare settings. However, we failed to show that this has any efficacy with regard to office diastolic BP, ambulatory BP or heart rate variability parameters. In addition, our dropout rate was higher than in previous studies conducted in laboratory or home settings, which suggests that caution is required in implementing isometric handgrip training in primary care settings.

The present study presents limitations that should be considered. The sample size did not allow for stratiﬁed analysis according to the medication used. This might have enabled comprehension of the mechanism(s) of BP lowering after isometric handgrip training. Generalizations of these findings to other populations (either those with advanced hypertension or other populations) must be made with care. The dropout rate in this study was higher than that we would have liked. It is not possible to assume that similar results would be observed among patients who dropped out of the program, and these data should be considered with caution. In addition, we did not do intention-to-treat analyses. Although without any statistically significant difference, the control group was heavier than the IHT group, and this needs to be taken into account. Lack of control regarding physical activity in both groups was also a limitation, although none of the patients engaged in any exercise programs. Lastly, other mechanisms for BP lowering after isometric handgrip training, such as baroreﬂex sensitivity, vascular measures or use of biomarkers, were not assessed.^27,32,38^

## CONCLUSION

Isometric handgrip training performed in a primary care setting reduced office systolic BP in hypertensive patients, whereas no effects were observed in relation to office diastolic BP, ambulatory BP or heart rate variability.
